# How to Educate Pregnant Women about Endocrine Disruptors?

**DOI:** 10.3390/ijerph17062156

**Published:** 2020-03-24

**Authors:** Steeve Rouillon, Houria El Ouazzani, Jean-Benoit Hardouin, Line Enjalbert, Sylvie Rabouan, Virginie Migeot, Marion Albouy-Llaty

**Affiliations:** 1 Health-Endocrine Disruptors-EXposome (HEDEX), INSERM-CIC1402, University Hospital of Poitiers, 86000 Poitiers, France; steeve.rouillon.c2i11@gmail.com (S.R.); houria.el.fellah.el.ouazzani@univ-poitiers.fr (H.E.O.); sylvie.rabouan@univ-poitiers.fr (S.R.); virginie.migeot@univ-poitiers.fr (V.M.); 2 Department of Public Health, BioSPharm Pole, University Hospital of Poitiers, 86000 Poitiers, France; 3 UMR CNRS 7285, IC2MP, 86000 Poitiers, France; 4 Faculty of Medicine and Pharmacy, University of Poitiers, 86000 Poitiers, France; 5 UMR SPHERE, Nantes, 86000 Poitiers, France; jean-benoit.hardouin@univ-nantes.fr (J.-B.H.); l.enjalbert@yahoo.fr (L.E.)

**Keywords:** health education, endocrine disruptor, pregnant women, educational posture

## Abstract

*Background:* Despite mediatization, only half of pregnant women are informed about endocrine disruptors (EDs). We wished to inquire about appropriate environmental health education procedures during pregnancy: Who, when, and how? *Methods:* The question stems from a comprehensive population health intervention research project. It includes qualitative studies aimed at constructing an educational program in environmental health and an accompanying assessment tool. The validation of a customized questionnaire (PREVED^©^ for Pregnancy Prevention Endocrine Disruptors) about the knowledge, attitudes, and practices (KAP) of pregnant women regarding exposure to EDs was carried out in a quantitative study. Results: Health education by a prenatal professional with communication skills should take place as early as possible, during the preconception period or early pregnancy, as part of individual consultation or group workshops. In order to customize the discourse and to develop women’s empowerment, concomitant presentation of the risks by the products used in each room and of previous solutions is recommended. *Conclusion:* Appropriate health education procedures on EDs should be done at every contact but taking the KAP of pregnant women into account first. We propose all educational actions should be accompanied by questioning of the KAP of pregnant women; for example, with questions from the PREVED^©^ questionnaire.

## 1. Introduction

Because of their transplacental passage, endocrine-disrupting chemicals (EDs) are environmental factors currently thought to affect the development of fetuses and young children following exposure during the in utero period, with long-term consequences for their future lives [1]. As a result, a significant number of pathologies and disorders are considered to be related to prenatal exposure to EDs: Low birth weight [2,3], prematurity [4,5], asthma and allergies [6], pubertal development disorders [7], congenital abnormalities [3], neurobehavioral disorders [8,9] and breast cancers [10].

In this context, the benefits procured from a reduction of pregnant women’s exposure to these molecules appear to be real. As a lack of information delays enlightened choices [11], the informing of pregnant women seems essential. While pregnant women are nowadays increasingly more widely informed, however, only 7% to 40% seem concerned [12], and few of them stop consuming products with EDs, such as cosmetics [13]. While numerous sources of information exist today (internet, television, and magazines cited by pregnant women) [14,15], they tend to target health professionals, such as educators [11,15,16]. According to professionals, information fails to penetrate due to missing informative tools [17] and the anxiogenic aspects of the topic [14,17].

However, information does not suffice to change practices [15]. Indeed, advice on physical activity or nutrition does not fit well with pregnant women’s needs, because it is not adapted or too anxiety provoking [18]. Practices are conditioned by psychosocial and socioeconomic characteristics [19]. Indeed, practices depend on attitudes, such as self-efficacy (or empowerment); cues to action; risk perception, which is associated to knowledge; and sociodemographic characteristics. Moreover, socioeconomic characteristics are correlated to empowerment levels [20]. These determinants are summarized in theoretical psychosocial health behavior change models, such as the health belief model [21,22]. The adaption of education to these determinants is a necessity [19]. Health professionals ought to perform educational tasks, but consultation time, educational tools, and psychosocial training are insufficient [23].

Among such tools, pamphlets are the most widely cited in the environmental education literature [24,25] because they summarize key elements [25]. However, they are merely informative and are not always adapted [26]. Moreover, pamphlets are overly complex, especially for poor health literacy-level persons. Other tools exist, such as serious games or videos. They have the advantage of being utilized outside consultation during lengthy waiting times [27,28]. Another way to educate outside the consultation time is through antenatal workshops during which parents share their knowledge, attitudes, and practices and develop new practical and emotional knowledge [29]. For example, a green cleaning party with a “do it yourself” method may be organized [25]. Given these different approaches, as part of interventional research on environmental health education, we tried to determine the most effective way of educating pregnant women on EDs: Who, when, and how? 

## 2. Methods

### 2.1. PREVED Project

Interventional research on environmental health education was carried out so as to construct an environmental health education program for pregnant women. It was called Pregnancy Prevention Endocrine Disruptors (PREVED). This project consisted in qualitative and quantitative studies. The study methodology is described elsewhere [14,22,30,31] and summarized in Table 1. Briefly, a review of the literature on theoretical models of health behaviors motivated our choice of the health belief model, which is organized around “risk perception” (severity and vulnerability), “belief in action” (through levers/barriers in adoption of a healthy behavior), and facilitators (sociodemographic characteristics) [21]. We also carried out 12 semi-structured interviews of pregnant women, and two focus groups of professionals. In both focus groups, we assessed the knowledge, attitudes, and practices (KAP) of pregnant women according to professionals and how to educate pregnant women on EDs [14,22,30]. These steps allowed us to construct an environmental health program [31] and its assessment tool: PREVED © questionnaire. The questions were aimed at assessing the efficacy and effectiveness of the program in terms of the knowledge, attitudes, and practices of pregnant women towards endocrine disruptor exposure.

### 2.2. Perception of Professionals on Who, When, and How to Educate Pregnant Women

The first focus group of professionals took place in March 2015 on the premises of the Faculty of Medicine and Pharmacy of Poitiers and the second in September 2015 during a training day. The target populations of the focus group are described elsewhere [22,30]. Briefly, it consisted in a student midwife, a pediatric nurse from the district office of maternal and childhood protection (Protection Maternelle et Infantile, the French PMI), a student in prevention psychology, a project leader at the French health care mutual, a project leader at a French association involved in health education and promotion, an organizer of health education workshops and a PhD student in environmental health [22] for the first focus group, and 11 perinatal health professionals working with an underprivileged population [30] for the second. Focus group participants were recruited by taking into account their involvement in the field of environmental health education (researchers, prevention actors, deciders, health professionals). The sample size of the focus groups was adapted to a qualitative approach, so it was able to provide both much information and free speech. Pregnant women participating in semi-structured interviews were recruited from medical records, taking age, gender, and type of housing into account in order to constitute a diversified population.

The question addressed to the groups we present in this article was: “How should we talk about perinatal exposure to endocrine disruptors?”. The focus groups lasted 90 and 62 min, respectively, and were recorded in the presence of an organizer (M.A.-L; C.M.) and an observer (J.A.; M.A.-L.). Idea saturation was sought. Content analysis with triangulation was the analytical method applied to this phase to select/organize the collected ideas in thematic trees.

### 2.3. Construction of the Assessment Tool (PREVED© Questionnaire)

In accordance with the validation method, we carried out a qualitative internal validation of the questionnaire assessing the efficacy of the health education program: A preliminary version was given to each contributor to the consortium, in order to select the dimensions to maintain, to add or to delete (and to reformulate questions, if needed). The order of questions and their mode of administration (auto- or hetero-administration) were discussed. A pre-test phase was then carried out to assess the acceptability of the questionnaire for an initial population of 30 pregnant women. Informed consent was obtained for this study. Metrological analysis was carried out and dealt with problems encountered when filling out the questionnaire: Frequency of missing responses and incoherencies. One question had too many of the above problems, another was inconsistent, yet another presented a problem in the wording, and three had unsuitable response forms. Then, a test phase was carried out and the questionnaire was administered to a second population of 300 pregnant or post-partum women to assess its reliability and building validity. In this phase, we determined whether the information provided for different groups of items and related to the same dimension could be summarized by new scores. We used Loevinger’s H coefficient for response coherence and Cronbach’s alpha coefficient for score reliability. A Cronbach’s alpha superior to 0.7 and an H coefficient superior to 0.3 were enough. The last step was the adjustment phase: Administration of the questionnaire to a third population of 30 pregnant women permitted its finalization.

Participants were pregnant women without complications and hospitalized women for whom delivery took place in a maternity unit with an uncomplicated delivery and with a healthy newborn. They were at least 18 years old and spoke French. They were recruited by clinicians, through leaflets in midwives’ offices in the three maternity units of the department, or on a social network. All women gave informed written consent. Their socio-demographic characteristics are detailed in a previous publication [14].

The sample size was defined according to the requirements of metrological analysis. Especially for the test phase, the sample size was also defined according to the number of dimensions explored by the questionnaire.

## 3. Results

### 3.1. Perception of Professionals on Who, When, and How to Educate Pregnant Women

#### 3.1.1. Who

**Figure 1:**
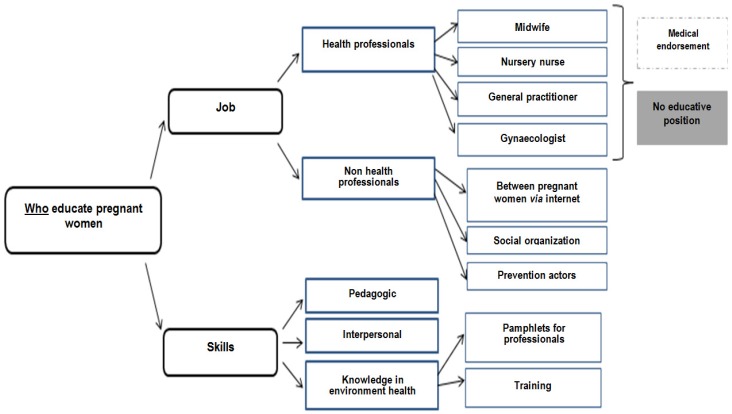
Perception of professionals on “who educate pregnant women”.

Findings on “who educate pregnant women” are presented in Figure 1.

Among professionals, different actors could educate pregnant women: Health professionals or not. Health professionals had greater medical endorsement then non-medical professionals. The verbatim were:

- “In the encounter with these young women, the midwife who travels to homes, who sees her in the preparation of childbirth, can be the means.”
- “PMI professionals, midwives and prevention workers who are used to doing what is called secondary prevention.”
- “Would the attending physician perhaps be suitable, it is the physician who perhaps sees the patient most often, [silence] it is not necessarily on the perinatal subject.”
- “Rather educate professionals who intervene before [pregnancy], especially gynecologists”
- “Information distributed by health professionals is highly listened to.”

However, more than job skills were discussed: Pedagogic, interpersonal, and scientific skills. The verbatim were:

- “It is true that health professionals are not necessarily equipped, so it would be a platelet almost for health professionals, to get in touch with people.”
- “In front of a professional they won’t dare ask questions…”
- “The gynecologist can provide a medical guarantee but I’m afraid that as it is not his role, that he delivers the information with all the weight of a doctor but without accompaniment.”

#### 3.1.2. When

**Figure 2:**
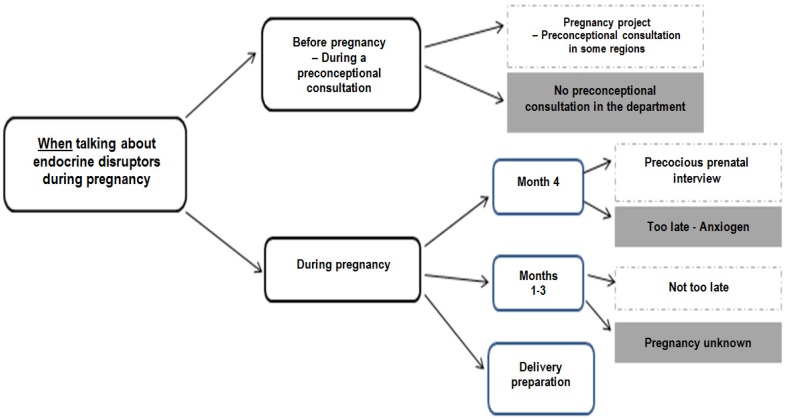
Perception of professionals on “when talking about endocrine disruptors during pregnancy”.

Findings on “when talking about endocrine disruptors during pregnancy” are presented in Figure 2.

Professionals thought that pregnant women should be educated before pregnancy or during the first four months in the prenatal interview. The verbatim were:

- “The time would be even before pregnancy, not necessarily when people are concerned about the issue, but the issue would be before.”
- “First introduce this topic, when women are planning pregnancy, perhaps more …because the goal is to prevent as soon as possible.”
- “I think the 4th month maintenance might be interesting, it’s a time when you really address the patient’s entire environment, you address her daily life, the environment in which she lives, her habitat, This could be a good time to talk about endocrine disrupters, they are quite open and we really talk about their whole environment at that time.”
- “The risk when you start, when you give this information at the fourth month, it’s true to say, well, all I’ve done before, that’s the limit” which can then cause ‘anguish’.”

However, it was thought that there are limits because it is a delicate period owing to possible miscarriage. The verbatim were:

- “The issue would be earlier, or at least in the first 3 months.”
- “Often the first three months are the time when there are no periods, the first ultrasound is the time when you don’t know too much, they don’t know if they are pregnant, it’s true it’s a bit complicated.”
- “The first 2-3 months, well, it’s true that this is a time when people are a little lost.”

Pregnant women could be educated at the end of the pregnancy when they have more time. The verbatim were:

- “There are prep classes at the hospital, and it’s true that we’re talking a little bit about bisphenol A, so we’re talking about glass baby bottles, all those things, after that it’s still little tips like that but it’s really not dedicated to that yet.”
- “They have time, they are on maternity leave.”

#### 3.1.3. How

**Figure 3:**
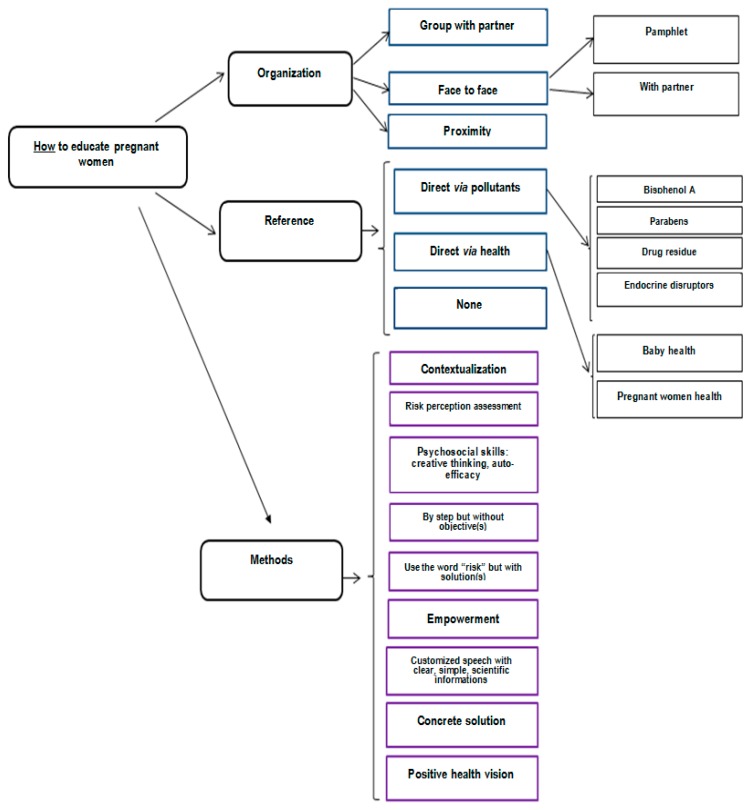
Perception of professionals on “how to educate pregnant women”.

Findings on “how to educate pregnant women” are presented in Figure 3.

For professionals, different tools help to educate, including pamphlets, interviews, speaking groups, and workshops. Each one has advantages and drawbacks. Pamphlets or notes in the maternity notebook permit easily delivery by professionals of simple, visual, and controlled information. However, information is not customized and could be anxiety provoking, as it is not adapted to the health literacy level because it is too scientific or complex. On the contrary, interviews permit customization of the message with adaptation to the health literacy level and questions from pregnant women, so it is less anxiety provoking. However, it is time-consuming for professionals. Workshops, which mix different people with different KAP and involve partners, permit the experience to be shared so are less anxiety provoking. However, as they are not individual, they cannot be customized, particularly for women for whom asking questions is difficult and so the recruitment of pregnant women is integral. Interviews and workshops are more costly strategies than pamphlets.

For professionals, the different methods to educate pregnant women on endocrine disruptor exposure are an educative position that highlights active listening (Table 2).

### 3.2. Construction of the Evaluation Tool (PREVED© Questionnaire)

The PREVED questionnaire consists of 33 questions [31] among which 8 should be used during interviews or workshops (Table 3). The metrics of the new questionnaire are presented in Table 4. These eight questions explore three dimensions:

- Knowledge about ED, composed of four questions about routes and sources of exposure, ability to name some ED molecules or families of molecules, and a definition of an ED. A catalogue of pictures illustrating sources of exposure helped the interviewer to ask questions.
- Attitudes as perception of risk with two components: Perceived severity and vulnerability. The created score is based on and adapted from the Perception of Pregnancy Risk Questionnaire [16]. It is composed of three questions with a binary and/or visual analogic scale.
- Practices as the perceived ability to reduce one’s exposure to EDs with a visual analogic scale.

We also created questions on the knowledge and application of solutions helping to reduce a pregnant woman’s ED exposure (Table 5). When the pregnant woman spontaneously suggests a solution, she is asked if she has only the idea, if she already applies the solution, or if she intends to apply it.

## 4. Discussion

This is the first study to investigate not the content of the message, but the context for educating pregnant women on EDs: who, when and how.

We found that different professionals could educate pregnant women on ED exposure but that health professionals (midwife, nursery nurse, and physician) seem to have more legitimacy. However, we found that interpersonal communication and pedagogic skills are more important than job type. These skills permit more space to be given to listening than to transmitting information, to not judge, to ask more open questions, and to give more space to experience than inform, thereby obtaining the best results in practices change [32]. Actually, suitable communication skills are taught to nurses [33] but not sufficiently to others health professionals, in particular physicians [34]. Indeed, counseling about lifestyle habits can be difficult for physicians depending on their own lifestyle, work self-efficacy and personality, job stress, and professional resilience [34]. One perspective could be to develop interventions (resilience workshops, cognitive behavioral training, mindfulness, and relaxation) that aim to improve professional resilience and influence the frequency of counseling in a positive direction [35]. So, our results defend the animation by trained health professionals of medical interviews or workshops.

Pre-conceptional interviews are the most frequent moment to educate chosen in our study. This result was also found in the literature [12,29,36]. In the absence of pre-conceptional interviews, professionals should promote the beginning of the pregnancy. However, the right moment largely depends on the temporal and psychological availability of pregnant women. For example, as women may hesitate to declare their pregnancy to their employer during the first months, their exposure during this period is difficult to avoid [15]. Finally, whatever the time of pregnancy when the topic of ED is discussed, the important thing is that it is. In this way, some authors recommend doing basic health improvement work, enhancing practice change, at every contact between a person and a health professional [37].

We found different contexts of education, with interaction (individual interview or workshops) or not (pamphlets). Pamphlets, if well-constructed, are a useful tool but are complementary to interviews and workshops. Individual interviews, with numerous advantages and less drawbacks, seem the more efficient, as has been found in the literature [38]. However, it takes time. On the contrary, workshops enable pregnant women to exchange information with other couples, taking the time to do so [29]. The personal experience is taken into close account. Through these exchanges, the knowledge, attitudes, and practices of pregnant women are revealed. In this way, efforts should be done on the integration of pregnant woman’s socio-demographic characteristics, knowledges, attitudes, and practices during every contact [30,39]. Our results support education with simple words, taking the time to listen to pregnant women’s representations, and questioning them. This result is in accordance with those from Haruty et al. [25]. Knowledge could easily be assessed by a pre- and post-test [36]. Practices could be assessed by simply and systematically asking what women have done about EDs in their house to reduce their use [15]. Attitudes are potentially the most complex to assess but could be done by risk perception assessment. The three dimensions could be assessed by an educational tool, as a part of the PREVED^©^ questionnaire, to help health professionals.

Based on this information, educators should customize speech using the word “risk” with “solutions proposed by the woman”, immediately highlighting that it is possible for her to reduce her exposure [30] in a positive vision. This vision excludes paternalism, moralizing, guilt-inducing, and being seemingly mandatory. It is an humanistic vision, described in the literature as preferable to an ideology of zero risk [40].

In environmental health, we defend the development of new ways of preventing risks by taking the local and societal context into account. Haruty et al. deem it advisable to value health-related changes in practices that will be accepted not only for reasons of personal satisfaction but also to reinforce the positive vision of information campaigns, and to achieve collective emulation favorable to global social change. To do this, it seems necessary to train health professionals to think in terms of salutogenesis and not only, as is usually the case, in terms of risks, thereby counteracting the disturbing aspects that we have noted [41]. This result is in accordance with the literature, especially owing to the growing demands of patients [42].

Several methodological aspects should be discussed in our study, such as our choice of using the HBM. In the literature, HBM has been used to both develop and assess prevention interventions and provides value in the exploration of both risk perception and cues to preventive action [21,43,44]. Thus, through the use of key constructs of the HBM, these studies were able to highlight the need to provide comprehensible and adapted information and education to pregnant women during prevention interventions. Eventually, an assessment of risk perception through perceived severity and vulnerability is a prerequisite to predict the probability of the adoption of healthy behaviors and eviction of inappropriate ones [45,46]. This is why HBM was suitable for our study.

Another methodological point is about the focus group. In this qualitative phase of the study, we aimed to collect views of every kind of professional involved in environmental health education in the context of constructing the program. The fact that only two focus groups were used was justified by the very important quantity of information easily provided by this kind of qualitative approach and was comforted by looking for the saturation of ideas. Regarding pregnant women’s KAP, the choice of semi-structured interviews was directed by the aim to individually collect the KAP of each pregnant woman. Thus, each pregnant woman was able to speak freely and was not influenced by another one, as it may have been the case if the focus group approach was chosen in this population and context.

## 5. Conclusions

Appropriate health education procedures on EDs should be done at every contact but as soon as possible and taking the KAP of pregnant women into account first. We propose all educational actions should be accompanied by questioning of the KAP of pregnant women, for example, with questions from the PREVED^©^ questionnaire.

## Figures and Tables

**Table 1:** Phases of the PREVED project.

Phases of PREVED Project	Aims	Period	References
Constitution of consortium	to involve researchers, prevention actors and deciders on the project	2014	
Review of the literature	to choose a behavior change model	2014–2015	
Interviews of 12 pregnant women	to explore dimensions for constructing the program	2015	[14]
Focus group of 7 health professionals	aim 1: to discuss how to educate pregnant women for construction of the program aim 2: to explore how to assess the program	03/2015	aim 1: result section aim 2: [30]
Focus group of 11 perinatal health professionals working with underprivileged population	aim 1: to describe knowledge and attitudes of professionals aim 2: to describe practices of professionals = to discuss how to educate pregnant women for construction of the program	09/2015	[30]
Cross-sectional study on 30 women	to pre-test of the questionnaire	07/2015	
Cross-sectional study on 300 women	aim 1: to describe KAP of pregnant women aim 2: to validate the questionnaire	08/2015-> 04/2016	aim 1: [14] aim 2: result section
Randomized controlled trial on 268 women	to test the efficacy and effectiveness of the program	04/2017-> 10/2020	Protocol: [31] Results: ongoing

**Table 2:** Methods to educate pregnant women and questions to ask on an educational role in comparison with a biomedical position.

Methods	Biomedical Position	Educative Position
To customize speech taking representations of pregnant women into account	“EDs are chemical products where exposure is associated with diseases”	“what are EDs for you?”
To present risk and solution in the same time	“As EDs are present in plastics, you should avoid plastics”	“you told me there are EDs in plastics, what could you do to avoid it?”
To use the word risk but in a positive vision	“You must avoid this product”	“what could you do about ED exposure to improve health?”
To highlight solutions that the pregnant women can use to decrease this exposure		“It is a very good idea to have chosen a glass bottle!”
To empower, to motivate that it is possible to change		“You told me you wanted the best for your baby, what about doing as well outside the house as you do inside the house?” [cooking]
To have concrete speech		“In your house….”
To respect steps	“You should throw away all your plastic boxes”	“You can do that this week and think about another way to do better the following week”

**Table 3:** Part of the PREVED© questionnaire that could be used in consultation or workshops.

Dimension/Questions	Possible Spontaneous Answers	Score
**KNOWLEDGE [40,5 points] -> to multiplicate by 2.4691 to obtain a score on 100**		
1. How could you imagine that chemical products which could degrade your health enter in your body or in your baby’s body?	Skin, Breathing, Eating, Drinking water, Through the placenta	0–5 points
2. According to you, what are the sources of exposure to ED that could degrade your health?		
*Outside wrapping*	Mineral water, Tap water, Fresh fruits and vegetables, Shower gel, deodorant, perfume, Day cream, makeup, Baby cream, Diaper-wipe, Drug, Household domestic, Home improvement products, Air ambient, Furniture, Toys, Candle, Incense, Interior perfume	0–22 points
*In wrapping*	Plastic bottle, Card bottle, Cans for drinks, Cans for food, Vacuum pack, Shrink-wrapped tray, Glass bottle	
3. If you have heard about EDs, could you name some?	Bisphenol A, Parabens, Phthalates, Pesticides, PCB, Flame retardant, Alkyl phenol, Nitrate, Phytoestrogen, Heavy metal, Phenoxyethanol *(1 point for bisphenol A and parabens; 0.5 for another proposition)*	0–6.5 points
4. How could you define an ED?	Hormonal molecule, Chemical molecule, Molecule produced by body, Molecule which alters body functioning, bacteria, Drug, Natural molecule	0–7 points
**RISK PERCEPTION [SEVERITY: 400 POINTS—VULNERABILITY: 1400 POINTS]**		
SEVERITY: 5. In general terms, the risk of endocrine disruptor exposure for pregnant women health is:	Nul (0 points)-Light (33 points)-High (100 points)	0–100 points
6. In general terms, how do you evaluate the risk of endocrine disruptor exposure during your pregnancy for…	*A baby, An adolescent, An adult* Nul……………………………….very high *	3 times 0–100 points
VULNERABILITY: 7. For each scale, make a mark for your vision of the risk about ED		
*For your health*	Nonexistent……………………………….very high *	0–100 points
*For your baby to be born prematurely*	Nonexistent……………………………….very high *	0–100 points
*For your baby to have a congenital malformation*	Nonexistent……………………………….very high *	0–100 points
*For your baby to be small for gestational age*	Nonexistent……………………………….very high *	0–100 points
*For your future adolescent to be obese*	Nonexistent……………………………….very high *	0–100 points
*For your children to have asthma*	Nonexistent……………………………….very high *	0–100 points
*For your children to develop an allergy*	Nonexistent……………………………….very high *	0–100 points
*For your children to have immunity problems*	Nonexistent……………………………….very high *	0–100 points
*For your children to have premature puberty*	Nonexistent……………………………….very high *	0–100 points
*For your children to have problems making babies*	Nonexistent……………………………….very high *	0–100 points
*For your children to have autism*	Nonexistent……………………………….very high *	0–100 points
*For your children to have behavioral problems*	Nonexistent……………………………….very high *	0–100 points
*For your children to have problems walking*	Nonexistent……………………………….very high *	0–100 points
*For your children to have cancer when adult*	Nonexistent……………………………….very high *	0–100 points
**BEHAVIOR [100 POINTS]**		
PERCEIVED ABILITY 8. Do you think you are able to avoid chemical products like ED which disturb your health?	No………………………….……….A lot *	0–100 points

* Scales of 10 cm.

**Table 4:** Metric proprieties of dimensions in the extract of the PREVED© questionnaire.

Dimension	Test Phase		Adjustment Phase	
	α-Cronbach	H Loevinger	α-Cronbach	H Loevinger
Route of exposure	0.54	0.38	Questions were not modified	
Source of exposure	0.61	0.16		
Knowledge of name	0.13	0.30		
ED Definition	0.72	0.32		
Perceived severity	0.84	0.35	0.97	NA (VAS)
Perceived vulnerability	0.95	NA (VAS)	0.84	NA (VAS)

VAS: visual analog scale.

**Table 5:** Solutions proposed by pregnant women when was asked “how could you act to reduce endocrine disruptors?” (open question).

General Lifestyle	Diet	Cosmetics and Hobbies	Hygiene
To have a healthy lifestyle	To choose a balanced diet	Do not use pesticides/fertilizers in your garden- To wear gloves when gardening	To regularly clean fridge
Do not smoke, to not use drugs, not drink alcohol-To avoid self-medication	To consume foods from organic farming or one’s garden or fresh unprocessed foods-To be careful of the origin of the products you buy	To avoid exposure to paints and products for work/do it yourself	To wash clothes before wearing for the first time
To regularly walk-To reduce the use of the vehicle-To live in the country	To breastfeed	To avoid makeup, coloring hair, nail polish/To reduce or stop consumption of cosmetics-To avoid scented cosmetics	To have good hand hygiene
To avoid walking near treated fields-To avoid polluted areas/living in an unpolluted environment	To Homemade prepare-To cook well the food	To prefer home-made, organic or natural cosmetics without paraben	To protect yourself when using chemicals household products (gloves, mask, etc.)
To aerate its habitat/Clean air vents in habitat-To avoid dusty atmospheres	To use caterer preparation	To prefer the purchase of cosmetics- personal hygiene products in (para)pharmacy	To reduce the use of chemical cleaning products
To prefer local products	To reduce the consumption of the canned foods or industrial foods or food additives or food containing GMOs	To prefer the liniment for children	To avoid using indoor perfumes, scented candles or incense, essential oils, sprays and aerosols or inhaling cleaning products or maintenance products without odor/fragrance
To protect against chemicals at work-To keep dangerous products out of children	To avoid eating foods that have been frozen	To use biological diapers	To prefer home-made or hypoallergenic cleaning products or detergents, or natural cleaning products (e.g., white vinegar) or products without bleach or ecological cleaning products
To check the labelling of cosmetic products, foods, processed drinks and medicines -To prefer all products with a label	To reduce consumption of meat or fish	To avoid synthetic fluff	To avoid wipes for children
To learn, read, learn about exposure sources	To consume filtered tap water	To avoid plywood furniture	
To reduce the use of plastic dishes, To Recycle, collect waste, To prefer selective sorting	To wash or peel fruits and vegetables		
To limit exposure to waves (phones, wi-fi)	To use set of glasses, glass containers- baby bottles glass-made or plastic containers without ED/To avoid food in plastic containers		
To pay attention to quality water	Do not use -To reduce the use of aluminum foil		
To harvest rainwater			
To avoid products containing bisphenols			
